# Fever of unknown origin: multidrug-resistant *Escherichia coli* presenting as infectious spondylodiscitis

**DOI:** 10.11604/pamj.2025.51.27.48065

**Published:** 2025-05-30

**Authors:** Emanuel Narvaez Gallifa, Eliseo Alejandro Aguillon Garcia

**Affiliations:** 1 Facultad de Medicina de Tampico 'Dr. Alberto Romo Caballero', Universidad Autónoma de Tamaulipas, Tampico, Tamaulipas, Mexico,; 2 Department of Infectious Diseases, Hospital Angeles Tampico, Tampico, Tamaulipas, Mexico

**Keywords:** Benign prostatic hyperplasia, bacteriuria, infectious diseases, cystoscopy, urine cultures

## Image in medicine

A 71-year-old man with a past medical history of benign prostatic hyperplasia and recurrent bacteriuria was referred to our infectious diseases department for persistent fever lasting 4 weeks following cystoscopy and two urine cultures growing ESBL-producing *K. pneumoniae* and *E. faecalis*. He did not take medications. Review of systems was positive for fever and general malaise. Vital signs were normal. Physical examination showed poor dentition and decreased breath sounds on the left. Initial laboratories showed leukocytosis (11,800/cm^3^), neutrophilia (9,620/cm^3^), and elevated C-reactive protein (CRP) 69.4 mg/L; urinalysis was positive for leukocyte esterase. Urine culture grew difficult-to-treat resistant P. aeruginosa. Chest X-ray was unremarkable; Computed Tomography (CT) scan showed air in the retroperitoneal space adjacent to the psoas muscle and interaortocaval adenopathies measuring up to 32 mm. Treatment began with ampicillin and was escalated to ceftazidime/avibactam plus polymyxin E. By day 6, he developed cramps, paresthesia, and pain in his lower extremities and a new left inguinal lymphadenopathy. Magnetic Resonance Imaging (MRI) revealed changes consistent with spondylodiscitis at T11-L1. Culture and biopsy of T11-L1 grew *E. coli* resistant to fluoroquinolones and aminoglycosides. Blood cultures grew MDR-ESBL-producing *E. coli*; repeat urine culture was negative. Additional infectious workup, including PCR assays for tick-borne pathogens and tuberculosis, was negative. Fever rose to 39°C, CRP to 127.7 mg/L, and procalcitonin to 1.30 ng/mL. Meropenem was initiated for 1 week, then de-escalated to complete a 6-week course. He underwent three lumbar irrigations. No medication-related adverse effects occurred. He recovered and was discharged.

**Figure 1:**
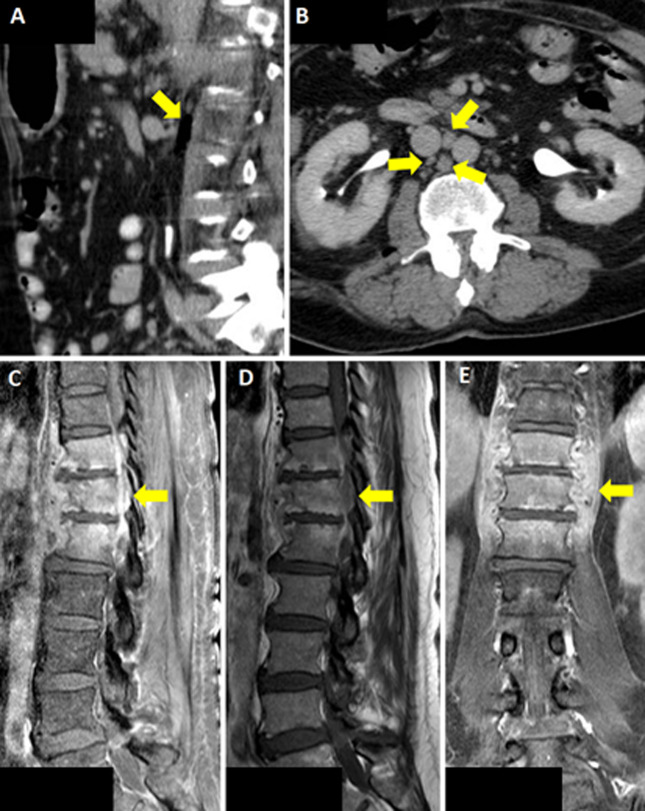
CT scan of the abdomen showing arrows pointing to: A) air in the retroperitoneal space; B) interaortocaval adenopathies; C,D) sagittal lumbar spine MRI, arrows pointing to T11-L1 with spondylodiscitis changes and associated paraspinal collection; E) coronal lumbar spine MRI, arrow pointing to T11-L1 with spondylodiscitis changes and associated paraspinal collection

